# Laparoscopic Resection of Advanced Colorectal Cancer in a Patient with Lumboperitoneal Shunt

**DOI:** 10.1155/2018/6826079

**Published:** 2018-11-14

**Authors:** Toru Imagami, Satoru Takayama, Yohei Maeda, Ryohei Matsui, Masaki Sakamoto, Hisanori Kani

**Affiliations:** Department of Surgery, Nagoya Tokushukai General Hospital, 2-52 Kouzouji-cho kita, Kasugai-City, Aichi 487-0016, Japan

## Abstract

A 78-year-old woman with lumboperitoneal (LP) shunt was diagnosed with advanced cancer of the ascending colon. Laparoscopic right hemicolectomy was performed without manipulating the catheter. The patient's postoperative course was uneventful, with no shunt-related complications or neurological deficit. The number of patients with cerebrospinal fluid (CSF) shunt who require abdominal surgery has been increasing. There are only few studies on laparoscopic surgery for patients with LP shunt, and the safety of pneumoperitoneum in the CSF shunt remains controversial. Consistent with other studies, we considered that pneumoperitoneum with a pressure of 10 mmHg has few negative effects. Our recommendations are as follows: (1) during colorectal resection, laparoscopic surgery can be performed without routine manipulation of the shunt catheter; (2) altering the location of the port is necessary to prevent both damage to the shunt tube during surgery and wound infection postoperatively; and (3) laparoscopic surgery is superior to laparotomy because it is associated with reduced surgical site infections and postoperative adhesions. However, laparoscopy should be performed at least 3 months after the construction of CSF shunt.

## 1. Introduction

Cerebrospinal fluid (CSF) shunts, such as ventriculoperitoneal (VP) and lumboperitoneal (LP), are widely used for the treatment of hydrocephalus. The development of shunt technology has contributed to improved patient survival rates, implying that an increasing number of patients will require abdominal surgery [1]. As the demand for minimally invasive surgeries increases, most abdominal surgeries are now being performed laparoscopically. Although there are several reports on laparoscopic surgery for patients with CSF shunt, laparoscopic colectomy for patients with CSF shunt has been rarely reported [2–4].

The main concerns regarding the use of CSF shunts during laparoscopic surgery are increases in the intracranial pressure, shunt dysfunction, shunt infection due to pneumoperitoneum, and surgical site infection. In a previous report, various management approaches for patients with CSF shunt during the perioperative period were undertaken, including extracorporeal clamping, external fistulation, and catheter removal [2]. However, no standard methods for the perioperative management of patients with CSF shunt currently exist. We performed a laparoscopic right hemicolectomy for a patient with cancer of the ascending colon and LP shunt. Here, we describe our clinical experience, along with a literature review.

## 2. Case Presentation

**Figure 1:**
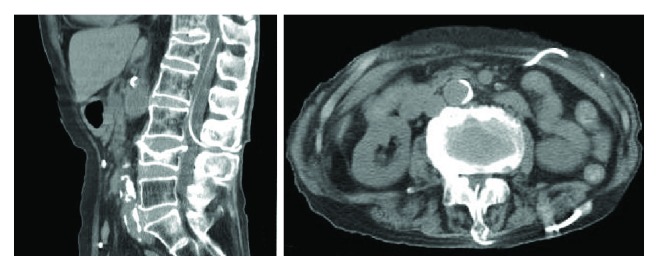
CT showed that LP shunt was routed from the subarachnoid space at the level of fourth and fifth lumber spines through the subcutaneous tissue of the left back and into the abdominal cavity.

A 78-year-old woman was referred for anemia investigation. She had a medical history of idiopathic normal pressure hydrocephalus (iNPH) and required LP shunt insertion. The patient underwent LP shunt insertion in 2016; however, the type of LP shunt tube used was unknown. Abdominal computed tomography (CT) showed a tumor in the ascending colon. Colonoscopy revealed cancer of the ascending colon with constriction. Regional lymph node metastasis was suspected, but there were no findings of distant metastasis. CT revealed that the LP shunt was routed from the subarachnoid space at the level of fourth and fifth lumber spine through the subcutaneous tissue of the left back and left flank and into the abdominal cavity (Figure 1). Although there was a certain risk of causing LP shunt-related complications during the perioperative period, her colorectal cancer was progressing, due to which we recommended a surgery.

**Figure 2:**
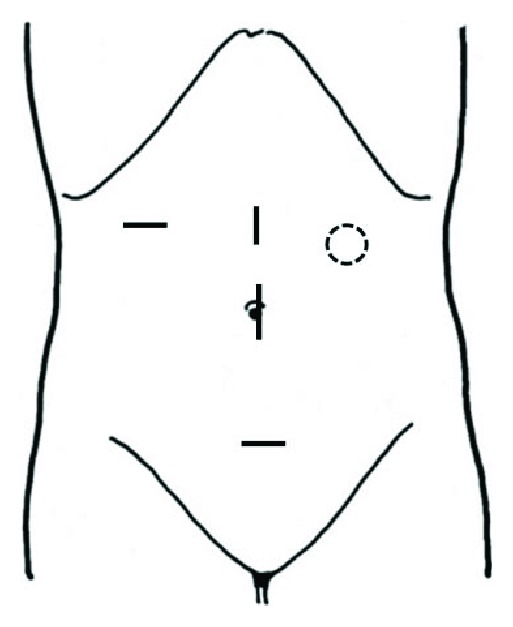
Port arrangement is shown by black lines. The umbilicus port is 12 mm, and the others are 5 mm port. The dotted circle indicates the site where the shunt tube is predicted to be inserted into the abdominal cavity.

**Figure 3:**
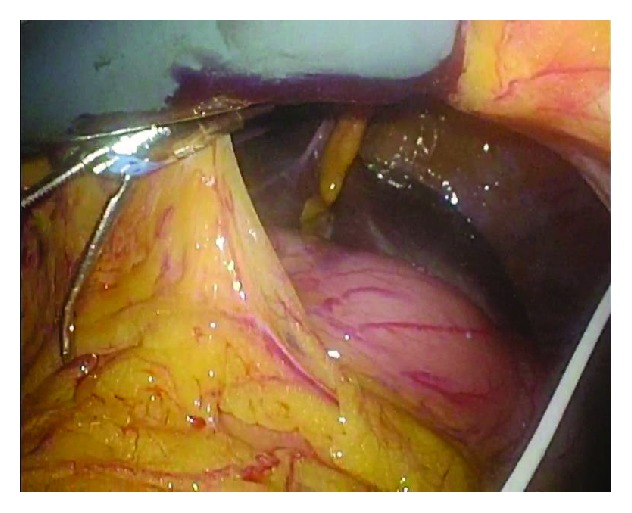
Intraoperatively, we could confirm the shunt tube and avoid damage.

During the perioperative period, no procedures, such as externalizing and clamping the shunt tube, were performed. Under general anesthesia, the patient was placed in the supine position. The first trocar was inserted through the umbilicus. After peritoneal insufflation using carbon dioxide, trocars were inserted, avoiding the left abdomen (Figure 2). Pneumoperitoneum was maintained at a pressure of 10 mmHg. The position of the shunt tube was confirmed in the abdominal cavity, and surgery was performed without any interference (Figure 3). Laparoscopically, lymph node dissection and mobilization were performed. Furthermore, minilaparotomy was performed to connect the incision of the upper abdomen to the umbilicus, and right hemicolectomy was performed with extracorporeal, ileocolonic anastomosis using a linear stapler. After the anastomosis, the abdominal cavity was cleaned with 2 L saline, and the wound was closed. No drain was inserted.

The patient's postoperative course was uneventful, with no shunt-related complications or neurological deficit. She underwent postoperative rehabilitation and was discharged 1 month postoperatively.

The patient's pathological diagnosis was primary double adenocarcinoma of the ascending colon, T4a, N0, M0, stage II and T2, N0, M0, stage I. Considering her performance status, she did not receive any adjuvant chemotherapy postoperatively. She remains well, with no findings of recurrence at 6 months postoperatively.

## 3. Discussion

In aging societies, the incidence of age-related diseases, such as iNPH, has been increasing and shunt interventions for iNPH have improved patient outcomes [5]. VP shunt is a common intervention for CSF drainage. However, LP shunt offers several advantages over VP shunt, such as no need for craniotomy and a lower incidence of shunt infection and malfunction [6, 7]. In a previous study, the efficacy of LP shunt was demonstrated, suggesting that it could be a first-line treatment option [7, 8]; this increase in the use of LP shunts may lead to a corresponding increase in the number of abdominal surgeries for patients. In another study, the presence of VP shunt did not pose an increased risk for postoperative complications in patients undergoing gastrectomy or colectomy [9]. However, reports on laparoscopic surgery in patients with LP shunt are fewer than those on surgery in patients with VP shunt [1, 7, 10, 11]. To the best of our knowledge, this is the first case of laparoscopic colorectal surgery in a patient with LP shunt.

The safety of pneumoperitoneum in CSF shunt is controversial. Currently, CSF shunts have a unidirectional valve to prevent backflow. Moreover, laparoscopic cholecystectomy without shunt manipulation has been recently performed [1, 10, 11]. Neale et al. reported that a disruption of shunt seals was noted at a pressure of 80 mmHg, with no leakage at 350 mmHg [12]. In another study investigating intra-abdominal pressures, the mean intra-abdominal pressure while standing is 20 mmHg and while coughing is 81.4 mmHg [13]. These results demonstrate that an abdominal pressure beyond the pressure of a typical pneumoperitoneum is not uncommon. In the anesthesiology literature, the impact of pneumoperitoneum on the function and cerebral blood flow of CSF shunt was evaluated using transcranial Doppler, with no deleterious effects observed [14]. Additionally, the laparoscopic-assisted catheter insertion of VP and LP shunts was first reported in 1993 and 1999 [15], respectively, and has become increasingly popular and results in better prognoses [16]. In addition to the insertion of the catheter, laparoscopic shunt revision is a safe and reliable technique [15]. Based on these findings, pneumoperitoneum with a pressure of 10 mmHg appears to have few negative effects [2]. LP shunt with a unidirectional valve was introduced in 1990s [6]. Therefore, LP shunts that were used after this period are expected to have a unidirectional valve.

However, it is a topic of concern that due to CO_2_ insufflation, the pneumoperitoneum may induce CSF shunt-related spread of cancer cells such as subcutaneous seeding or retrograde metastasis [3, 4]. Recently, long-term outcomes of large, randomized trials comparing open and laparoscopic surgeries for colorectal cancer demonstrated the noninferiority of laparoscopic surgery for treating T4 tumors and an actual incidence of port-site metastasis of approximately 1%, which is not significantly different from wound recurrence rate after open colorectal surgery [17]. Lee et al. suggested that the surgical technique used plays a larger role in the development of port-site tumors than that played by CO_2_ insufflation of the pneumoperitoneum [18]. Therefore, traumatic handling of tumors and inadvertent contact to shunt tube should be avoided to prevent tumor implantation. Regarding retrograde metastasis, a unidirectional valve seems to be expected to prevent retrograde metastasis of cancer cells as well as CO_2_. Conversely, Nawashiro et al. reported a case of subcutaneous seeding of pancreatic cancer along the fistula [19]. Particularly, in advanced cancer patients with CSF shunt, careful observation after surgery is considered necessary.

Li and Dutta suggested that there is a minimal risk of VP shunt malfunction or infection among patients undergoing routine abdominal and urologic surgeries [20]. Laparoscopic surgery without catheter manipulation, such as tube clamping or externalization, has also become a routine. Surgical site infection leading to shunt infection is a risk of laparoscopic colectomy as well as open laparotomy. In the research performed by the Japanese Clinical Oncology Group, complication rates for laparoscopic colectomy were 3.6% for anastomotic leakage and 5.3% for surgical site infection [21], which were comparable and lower, respectively, than those for laparotomy [21]. For shunt infection, laparoscopic surgery is superior to laparotomy, and except in high-risk cases of intra-abdominal infection, routine tube clamping and externalization are unnecessary.

To prevent the spread of infection to the shunt tube at the surgical site, altering the location of the port is required [10, 11]. In such cases, when the shunt catheter is inserted into the left abdomen, the port should not be located in the left abdomen, as shown in Figure 2.

The major complications of LP shunt are catheter obstruction and migration [6]. Postoperative adhesions and scar tissue have been reported to cause shunt obstruction and dysfunction [22, 23]. Wang et al. reported that most complications occur within 3 months of the surgery [6]. Therefore, laparoscopic resection to reduce postoperative adhesions may be superior to laparotomy. Furthermore, additional abdominal surgeries should not be performed within 3 months of CSF shunt construction.

In the Japanese guidelines for iNPH, LP shunt is considered unsuitable for patients with a lumbosacral decubitus ulcer [24]. During the perioperative period, patients are likely to develop decubitus ulcers. Although measurements against the development of decubitus ulcers are important in all patients, patients with LP shunt require additional attention.

In our case, the pathological diagnosis was T4a, N0, M0 with likely recurrence of peritoneal dissemination. There are no published studies on whether the effects of LP shunt can be maintained in case of peritoneal dissemination. We believe that it is necessary to consider our patient's clinical course as a rare case of advanced colorectal cancer with LP shunt.

This report describes a successful case of a patient with colon cancer and LP shunt who underwent laparoscopic resection without manipulation of the shunt tube. This is a single case report, which is a limitation of this study. Taken together, the establishment of surgical methods for patients with LP shunt is desirable due to the projected increase in the number of similar cases.

## 4. Conclusion

This report of a patient with advanced colon cancer and LP shunt describes a laparoscopic right hemicolectomy without manipulation of the shunt tube. There were no shunt-related complications. Our recommendations are as follows: (1) during colorectal resection, laparoscopic surgery can be performed without routine manipulation of the shunt catheter; (2) altering the location of the port is necessary to prevent damage to the shunt tube during surgery and wound infection after the surgery; (3) laparoscopic surgery is superior to laparotomy in reducing surgical site infection and postoperative adhesion, but laparoscopy should be performed at least 3 months after the construction of the CSF shunt.
